# Curiosities of Digestion

**Published:** 1886-07

**Authors:** 


### CURIOSITIES OF DIGESTION.

The once celebrated Arbuthnot insisted that the human stomach is a kitchen, while Dr. Beaumont, in his experiments on St. Martin, whose stomach was seen plainly for a long time during hours of digestion through an orifice made by a gunshot wound, showed that the organ was sort of a dining-room. Two recent experimenters have made the stomach a study, and now the question arises : What fluid really agrees with it? Signor Ogata of Italy experimented on a dog, and reached a fair conclusion as to the effects of certain beverages on human beverages generally. He took advantage of a stomachal fistula, which afterwards healed. He found that water, tea and coffee in moderate quantities have no effect on digestion, and hence the common idea not to drink water during a meal is fallacious.

Beer, wine and brandy retard the action of the gastric juice until absorption takes place. Sugar makes digestion difficult, while salt helps it. A surprise is in reserve. Dr. Cheltsoff, according to that high medical authority, the London Lancet, recently experimented on the effects of the best known bitter extracts, and found that the "pure bitters usually prescribed for dyspeptics or persons suffering from other and less serious digestive ailments, instead of stimulating, actually depress the stomach and injure digestion. They do not aid, but retard, the flow of the gastric juice. In large doses that indispensable agent of digestion is actually diminished, while small doses bring, as the doctors say, "a slight and transitory increase" of the juice. Some of the bftter extracts increase the secretion of bile, while others have no effect, but in nearly all cases the result is either unsatisfactory or injurious. This is bad news for aged ladies who take to their morning bitters as elixirs of life, and for venerable gentlemen who delight to have their cocktails dashed with bitter extracts as a promoter of longevity and as an assistant eye-opener. "Bring me my boots and my bitters" will cease to be a matitudinal order, if Dr. Cheltsoff be extensively read. Men do not need bitters to keep healthy any more than women. Exercise in the open air, cessation from work when mind or body grow the least weary, regular hours, with plain, mixed diet.

